# When they just don’t sleep: differential impacts of reduced child sleep on depression, anxiety, and stress among caregivers of children with and without neurogenetic syndromes

**DOI:** 10.3389/fpsyt.2024.1352881

**Published:** 2024-04-19

**Authors:** Kimberly Gálvez-Ortega, Kristine Marceau, Dan Foti, Bridgette Kelleher

**Affiliations:** ^1^Department of Psychological Sciences, Purdue University, West Lafayette, IN, United States; ^2^Department of Human Development and Family Science, Purdue University, West Lafayette, IN, United States

**Keywords:** child sleep, sleep duration, neurogenetic syndromes, typically developing, caregiver, mental health, depression, stress

## Abstract

**Introduction:**

Children with neurogenetic syndromes commonly experience significant and pervasive sleep disturbances, however, associations with caregiver mental health remains unclear. Previous studies have linked sleep disturbances with increased caregiver depression in typically developing populations, and heightened caregiver stress among neurogenetic populations. The present study expands on findings by exploring the longitudinal association between child sleep duration and caregiver mental health (depression, anxiety, stress) throughout development (infancy to school-aged children) in dyads with and without a child affected by a neurogenetic syndrome.

**Methods:**

Participants were drawn from the Purdue Early Phenotype Study, including 193 caregivers (Age: *M* = 34.40 years, *SD* = 4.53) of children with neurogenetic syndromes (Age: *M* = 40.91 months, *SD* =20.72) and typically developing children (n = 55; Age: *M* = 36.71 months, *SD* = 20.68). Children in the neurogenetic group were diagnosed with Angelman (n = 49), Prader Willi (n = 30), Williams (n = 51), and Fragile X (n = 8) syndromes. Caregivers completed assessments every six months up to child age three, and annual assessments thereafter. Child sleep duration was measured using the Brief Infant Sleep Questionnaire, and caregiver internalizing symptoms were assessed using the Depression, Anxiety, Stress Scale. Multilevel models were conducted to examine caregiver depression, anxiety, and stress in relation to child sleep duration at both between- and within-person levels, with child age as a moderator.

**Results:**

Results indicated a between-person effect of child sleep duration on caregiver depression (i.e., differences between families) and a within-person effect on caregiver stress (i.e., change over time) in the full, combined sample. These effects were not maintained when examined separately in neurogenetic and typically developing groups, except for a between-person effect on caregiver stress in the typically developing cohort. Moderating effects of child age were significant for depression and stress only in the typically developing cohort.

**Discussion:**

In summary, persistent child sleep disruptions were linked to exacerbated caregiver depression across the sample, while acute child sleep disruptions exacerbate caregiver stress within dyads over time. These findings emphasize the importance of addressing child sleep to enhance caregiver wellbeing and has potential relevance for a wide range of neurogenetic syndromes.

## Introduction

Sleep disturbances in children are common, such that approximately 25% of children experience clinically significant sleep problems (1), including daytime sleepiness and short sleep duration (2, 3). In addition to the direct consequences of sleep disturbances on child health (e.g., increased depression and anxiety risk, neuropsychological functioning, and behavioral problems including inattention, aggression, and increased injury; 4–7 8, 9), child sleep disturbances are also associated with *caregiver* health, particularly symptoms of depression and other mental health outcomes (10–13). This association may be particularly problematic in populations affected by elevated sleep problems and caregiver strain, including families of children with neurogenetic conditions affiliated with atypical sleep. The present study examined the degree to which child sleep and caregiver well-being unfold over time in neurogenetic populations. The broader aims are twofold. First, this work may inform optimal therapeutic targets for interventions among neurogenetic syndrome (NGS) families. Second, studying how these patterns unfold in a highly saturated, high-risk sample may inform broader understanding of how family sleep dynamics unfold, with potential applications among the general population.

The association between child sleep and caregiver mental health is well established in the literature (14–16). For example, a longitudinal cohort study examined sleep patterns in 483 infants two weeks after birth, and 2, 4, 8, 12, and 24 months later, as well as caregiver levels of depression and stress at 24 months (13). Per caregiver report, approximately 10 to 20% of infants exhibited sleep problems across waves, with 6.4% of infants demonstrating problems in three or more observations. Importantly, persistent sleep problems accompanied with crying and fussing behaviors predicted later levels of depression and stress in parents (13), suggesting repeated sleep problems in children may have an impact on caregiver mental health. These patterns may also emerge bidirectionally; in a recent large-scale meta-analysis of 3,009 participants’ data from the Environmental influence on Child Health Outcomes (ECHO) study, caregiver stress and child medical conditions were both uniquely found to predict concurrent child sleep outcomes. However, given the cross-sectional nature of these data, it is not possible to determine the directionality of these effects.

Complementary findings have emerged from child sleep intervention studies, which inform potential directionality by determining the degree to which improving sleep might similarly improve caregiver wellness. Indeed, several interventions targeting child sleep also have demonstrated secondary effects on caregiver mental health symptoms. For example, one study of caregivers of 8 to 12-month-old infants showed that, after completing a sleep intervention program, the caregivers reported improvements in their child sleep behaviors and decreases in their own depressive symptoms (10). Similarly, a study of mothers of 6 to 12-month-old infants with severe sleep difficulties enrolled in a child sleep intervention group and reported decreased depressive symptoms following treatment, and symptom improvements remained at a four-month follow-up (11). Together, these studies suggest that sleep is a modifiable risk factor that may contribute to caregiver well-being.

The longitudinal pervasiveness of a child’s sleep challenges also appears to impact caregiver outcomes. As an extension to Hiscock and Wake’s (11) study, Lam and colleagues (12) conducted follow-up assessments with mothers that participated in the infant sleep study three to four years post-treatment, reassessing both children sleep patterns and maternal depression symptoms. Researchers found children’s persistent or reoccurring sleep problems from infancy to early childhood (3 to 4 years of age) predicted higher depressive symptoms in mothers. Notably, the reverse association was not detected: depression symptoms endorsed by mothers when their infants were 6-12 months old did not predict subsequent sleep difficulties when their children turned 3 to 4 years old. These longitudinal findings suggest that depressive symptoms in caregivers may be an outcome of child sleep problems, rather than a cause.

Given the robust associations between child sleep and caregiver well-being in population samples, it logically follows that caregivers whose children are predisposed to atypical sleep may be particularly affected. Indeed, many neurogenetic syndromes (NGS) are associated with severe and pervasive sleep disturbances (17, 18) that emerge early in children’s development (19). One of the most commonly cited NGS associated with sleep challenges is Angelman syndrome, a NGS associated with absent speech, severe intellectual disabilities, seizures, and a unique behavioral profile that often includes a happy demeanor and laughter (20). Sleep challenges are one of the most common issues reported by Angelman caregivers; a study of 49 caregivers of children ages 2-26 years indicated that relative to age-matched controls, “Angels” regularly slept less than eight hours during the night (70.27% versus 9.63% in age-matched TD children), woke up two or more times (62.16% versus 6.83% in TD children), had difficulty falling back asleep (56.76% versus 4.82% in TD children), took longer than 30 minutes to fall asleep (32.43% versus 6.61% in TD children), and screamed at night (18.92% versus 5.49% in TD children; 21). Abel and Tonnsen (19) found that infants and toddlers with Angelman syndrome exhibited 3 times longer night wakings than non-NGS controls, with 50% percent of caregivers endorsing some degree of sleep problem in their child. Given these challenges, sleep is rated one of the top priorities for treatment among Angelman syndrome caregivers, particularly among families of young children (22).

Sleep challenges are also present among other NGS. For example, 97% of caregivers of school-aged children with Williams syndrome report their child experiences frequent sleep interruptions, with more than two awakenings per night, and 60% exhibit sleep restlessness (23). These patterns appear to emerge early in development among people with Williams syndrome, with 61% of infant and toddler caregivers reporting sleep problems (19), which include shorter sleep duration and reduced sleep efficiency (24). Among children with Prader Willi Syndrome, 52% of caregivers report that their child exhibits night wakings, relative to 27% in TD controls (25); however unlike in Angelman syndrome and Williams syndrome families, a minority (16%) of caregivers of infants and toddlers report sleep problems (19). Among caregivers who participated in a survey of family needs associated with fragile X syndrome (n=1,295), 32% reported current child sleep challenges, 84% endorsed at least two unique sleep problems, and 47% (males) and 40% (females) were taking sleep-focused medication; infants with fragile X syndrome have been similarly reported to exhibit shorter sleep duration and reduced sleep efficiency relative to controls (24). Together, these studies demonstrate the pervasiveness of sleep challenges across a variety of NGS groups, as well as variability in the developmental emergence of challenges across groups.

Given that sleep problems are common and relatively severe across many NGS, families affected by neurogenetic syndromes provide a uniquely informative context in which to explore the associations between disrupted child sleep and caregiver mental health outcomes. Two studies to date have examined this relation in families affected by NGS. The first study conducted by Richdale and colleagues (26) explored caregiver-reported stress (via the Parenting Hassles Scale) and sleep issues in children with fragile X syndrome ages 3 to 19 years. This cross-sectional study found that caregivers who reported problematic sleep patterns (e.g., night awakenings, settling difficulties) for their child with fragile X syndrome had significantly greater levels of stress compared to parents that reported no problematic sleep patterns. A second study by Goldman and colleagues (27) explored the relationship between offspring sleep disturbance and caregiver mental health within in children and adolescents with Angelman syndrome ages 2 to 16 years. Over a 28-day follow-up period, the researchers assessed caregiver stress (Parenting Stress Index Short Form) and offspring sleep behaviors (actigraphy, polysomnography), and caregiver report (Children’s Sleep Habits Questionnaire). In line with the previous study, findings indicated that total variability in child sleep predicted higher caregiver stress (27). These two studies highlight that similar to TD populations, child sleep challenges are associated with a negative impact on caregiver mental health; however, the directional nature of these associations is not well defined.

A notable caveat to this literature is the elevated mental health and stress challenges that many NGS caregivers experience for reasons that stem beyond their child’s sleep. Although many NGS caregivers are well-adjusted (28), caregiver stress is common across NGS, due to increased medical appointments, financial burden, injury, child challenging behaviors, employment disruption, isolation, reduced parenting self-efficacy, and stress (29–31). Some NGS groups also experience unique caregiver vulnerabilities. For example, although many NGS are not inherited, genetic carriers of the *FMR*1 premutation, which expands to cause fragile X syndrome in a subset of offspring, demonstrate greater stress and mental health symptoms (32), which are associated with their own genetic architecture as a “carrier” (32, 33). Maternal carriers’ mental health and stress have also been linked to child behavioral symptoms (34, 35), demonstrating the complex, bidirectional system that likely places NGS families at enhanced risk for challenges.

These examples highlight the unique importance of studying child sleep and caregiver impact in the high-risk model of NGS families: in addition to the child being genetically predisposed to disrupted sleep, the caregiver is situationally (and for fragile X syndrome, sometimes genetically) predisposed to greater stress and mental health challenges. In this way, NGS families offer a powerful “saturated risk model” for understanding how – when both child sleep and caregiver wellness are at risk – one might influence the other over time. This knowledge has potential to not only improve outcomes for NGS families, but also inform how family dynamics may unfold in other high-risk groups with genetic or environmental vulnerabilities related to child sleep and caregiver well-being.

As these studies demonstrate, a wealth of literature has documented the importance of childhood sleep on family well-being, and the challenges NGS families face in both child sleep and caregiver wellness. However, there are three primary gaps limiting understanding of how to best support caregivers with NGS in addressing these challenges.

First, child sleep is a dynamic process that changes rapidly over time in early development, even among non-NGS groups. Indeed, whereas sleep patterns in adulthood are relatively stable, sleep patterns change dramatically within the first few years of life across infancy and early childhood (36). For example, in a longitudinal study of sleep patterns in TD children conducted by Quach and colleagues (37), sleep problems were more common at age 4 to 5 (4.3% severe, 8.7% moderate, 20.6% mild) and less common at age 6 to 7 (1.9% severe, 3.8% moderate, 7.0% mild). On the other hand, studies have found that sleep disturbances for some children do not always resolve with age (38–40) and variations in sleep duration may increase as children reach school-age years (38). Thus, examining within-person changes in sleep over time – in addition to examining group-level dynamics – is important to accurately define how sleep unfolds within families.

Second, sleep challenges are relatively common, both in TD and NGS populations. A previous study comparing sleep in NGS groups to non-NGS infants and toddlers indicated high rates of parent sleep concerns in both groups, with 38% of TD and 41% of NGS caregivers perceiving their child has having at least some sleep problems (19). Interestingly, in these same cohorts, only 17% of TD infants exhibited sleep durations that were atypical based on national guidelines (i.e., National Sleep Foundation; 41), compared to 31% of NGS infants. Although these results were reported in small samples, they suggest that NGS and non-NGS families both perceive sleep challenges at similar rates in early childhood, and perceptions of sleep challenges, and the degree to which these perceptions align with national standards, may differ across groups.

Third, the directional nature of family sleep dynamics is complex, and without a clear understanding of longitudinal sequelae, designing effective interventions is difficult. As such, a key next step is to explore the association between child sleep disturbances and caregiver mental health within a *long-term longitudinal* framework, which may shed light on the time course of these associations (e.g., acute versus chronic change in caregiver stress and mental health symptoms), as well as how this association is shaped by developmental changes in child sleep. For example, one possibility is that the effect of child sleep disruptions on caregiver mental health shifts over time as the child ages and their sleep challenges change. Quach et al. (42) reported that late bedtimes were associated with worse maternal mental health in 6 to 7 year olds, but not among 8 to 9 year olds. It is also possible that *persistent* disruptions of child sleep may have distinct effects from more acute, short-term disruptions of child sleep, as suggested by past longitudinal studies in which repeated reports of crying and fussing predicted greater caregiver depression and stress (13). To date, however, longitudinal studies have not probed these associations in NGS groups, limiting understanding of how these patterns may or may not extend to families in which the child is genetically predisposed to disrupted sleep.

The present study addresses these gaps by examining dynamic, longitudinal patterns of child sleep and caregiver mental health in a large cohort of families affected by NGS. These data were drawn from Phase I of the Purdue Early Phenotype Study (PEPS), a longitudinal caregiver-report study of early development across children with and without multiple NGS who contributed data between 2016 and 2021; data continue to be collected to date. This cohort provides a ‘saturated’ sample to study the consequences of poor child sleep on caregiver mental health given the high rates of both child sleep challenges and caregiver mental health needs in NGS communities. A portion of sleep data from this cohort have been published previously (n=80; Abel & Tonnsen); this cross-sectional study reported initial sleep problems in 41% of NGS participants, with 29% of children demonstrating abnormal sleep duration relative to national guidelines.

Here, we build upon these prior analyses by examining associations between child sleep disruptions and caregiver mental health in an expanded, longitudinal sample of 193 participants who contributed 718 observations during Phase I. Similar to Abel and Tonnsen, we operationalized child sleep disturbance as atypical *sleep duration.* We considered caregiver mental health by measuring self-reported symptoms across three clinical dimensions of internalizing psychopathology: depression, anxiety, and stress. Examining all three dimensions allowed us to test whether effects of child sleep disruptions were clinically specific or more general across the internalizing spectrum.

Specifically, we used mixed effects longitudinal models to determine (a) the degree to which child sleep duration predicted caregiver mental health symptoms of depression, anxiety, and stress, at both the between- and within-caregiver levels, (b) the effects of child sleep duration on caregiver mental health change over the course of early life development (i.e., child age, from infancy to school-age children) with a moderation of child age, and (c) whether these associations would be observed across the entire combined group and its sub-cohorts, including the NGS cohort (i.e., ‘high severity’ subgroup regarding child sleep) and the TD cohort (i.e., ‘low severity’ subgroup regarding child sleep).

We hypothesized that across the combined and sub-cohorts, there would be a significant, *between-caregiver* main effect of child sleep duration on caregiver mental health symptoms, such that caregivers of children that generally have shorter sleep durations across the study period would report greater symptom severity. We also hypothesized complementary *within-caregiver* effects, such that acute disruptions of child sleep—occasions when the child slept less than their own average—would be associated within increased caregiver mental health symptom severity. As exploratory analyses, we also tested whether the associations between child sleep duration and caregiver internalizing symptoms would be moderated by child age using between-family effects (i.e., comparing the effects of younger versus older children across families) and within-family effects (i.e., as children increase in age over the course of the study).

## Method

### Participants and procedure

Participants were drawn from the first phase PEPS (19, 43, 44) collected between 2016-2021. As part of the PEPS protocol, families were recruited via nationwide web-based support groups, syndrome research registries, and social networks, such as Angelman Syndrome Foundation and Registry (www.angelman.org) and Williams Syndrome Association and Registry (www.williams-syndrome.org/registry). Recruitment, consent, and procedures for this study were approved by Purdue University Institutional Review Board. To participate in PEPS, caregivers were required to primarily speak English and to be the biological parent of a child with NGS. Families were compensated for their time for completing each assessment. The frequency of family assessments was based on child age at the time: through child age 3, caregivers completed assessments every 6 months; after age 3, caregivers completed assessments annually. As part of their participation, caregivers completed a variety of web-based surveys related to child development and caregiver functioning, including measures on child sleep and mental health outcomes.

A total of 210 families completed assessments during Phase I of PEPS. The current analyses focused on a subgroup of 193 families with at least one observation of child sleep duration data (49 Angelman syndrome, 30 Prader Willi syndrome, 51 Williams syndrome, 8 fragile X syndrome, and 55 TD). This subgroup of families yielded a total of 718 observations (471 NGS, 247 TD), with the number of observations ranging between 1 to 7 per family, as detailed in **Table 1**.

**Table 1:** Number of Assessments Completed Across Cohorts.

Assessments Completed	NGS	TD	Total
1	20	3	23
2	27	5	32
3	22	7	29
4	29	5	34
5	26	16	42
6	13	19	32
7	1	0	1

## Measures

### Child sleep

Sleep patterns among offspring were assessed using the Brief Infant Sleep Questionnaire (BISQ)-Brief across phase 1 of the study. The BISQ-Brief (45) assesses sleep patterns in infancy to early childhood (i.e., How much does your child spend in sleep during the night)? and parent perceptions of child sleep (i.e., Do you consider your child’s sleep a problem?; α = .57). The current analyses utilized the following 2 items from the BISQ: “How much time does your child spend in sleep during the NIGHT (between 7 in the evening and 7 in the morning?” and “How much time does your child spend in sleep during the DAY (between 7 in the morning and 7 in the evening)?” Sleep duration was collected in minutes, and numeric responses to both items were added to calculate the number of minutes children slept within a 24-hour period, such that higher values indicated longer sleep duration and lower values indicated less sleep duration within a 24-hour period. While other sleep measures were also included in the data collection process, the BISQ-Brief was selected over other measures because (a) the BISQ has been validated against parent report sleep diaries as well as actigraphy (45), (b) the BISQ was collected at each time point, and (c) is typically administered to parents with younger children, which was an excellent fit for the age range of our child sample. In our sample, only one data point for child sleep duration was removed, given the recorded sleep duration was 1695 minutes (~28 hours), which is not plausible.

### Caregiver mental health outcomes

Caregiver mental health outcomes were operationalized in the context of self-reported depression, anxiety, and stress symptom severity on the Depression Anxiety Stress Scales (DASS-21; 46). The DASS-21 is a reliable and validated measure that has been used to assess internalizing symptoms across clinical (47) and non-clinical samples (48). The measure is comprised of three separate subscales (i.e., depression, anxiety, and stress) with seven items each, yielding a total of 21-items. Caregivers rate each item using a 4-point scale (*0 = Never, 1 = Sometimes, 2 = Often, 3 = Almost Always*) indicating how frequently they experienced the specified symptom over the past week. The DASS-21 was designed according to the tripartite model of internalizing symptoms: Specifically, the Depression subscale assesses low positive affect, which is relatively clinically distinct from Anxiety and includes feelings of hopelessness and lack of interest or enjoyment (i.e., I felt like life was meaningless; α = .87). The Anxiety subscale assesses physiological hyperarousal, which is relatively clinically distinct from Depression and includes panic-like symptoms (i.e., I felt I was close to panic; α =.73). The Stress subscale captures more general, non-specific negative affect that is clinically shared across depression and anxiety disorders (i.e., I found it hard to wind down; α = .84). Scores for the Depression, Anxiety, and Stress subscales are calculated by adding the scores for each relevant item. A higher score on a DASS-21 subscale indicates increased mental health severity (or worse mental health). In the present sample, prominent skewness was observed for scores on the Depression and Anxiety subscales. To address this skewness, subscale scores were winsorized and later transformed using the square root. Scores on the Stress subscale were winsorized, but only in the TD cohort due to prominent skewness (detailed in **Appendix B**).

### Analytic strategy

To assess how the association between child sleep duration and caregiver mental health symptom severity changes over the course of development (from infancy to school-age children), we conducted mixed effects models using the Mixed Procedure (PROC Mixed) in SAS version 9.4. Analyses were completed for each group independently (i.e., NGS cohort, TD cohort) and as a combined cohorts (i.e., combined NGS and TD groups). Several steps were taken to build the models of the current study. First, an unconditional model was calculated for each mental health outcome (i.e., Depression, Anxiety, and Stress subscales). Unconditional models include the intercept without predictors, yielding the intraclass correlation coefficient (ICC) for each variable of interest. ICCs were used to assess the proportions of variance in Depression, Anxiety, and Stress scores that are attributable to between-person variance [(*σ^2^_u0_*)/the total variance (*σ^2^_u0_* + *σ^2^_e_*)] and within-person variance (1 - ICC). Each unconditional model provides a reference point to compare changes in between- and within-person variances when predictors were added to the mixed-effects models, such that child sleep and child age in months.

Following the unconditional models for all mental health outcomes, additional unconditional models were conducted for child age, caregiver age, and child sleep to determine how to treat these variables in the hypothesis-testing analyses. Across cohorts, ICC analyses revealed that child age variances were largely accounted by within-person differences (71.10% in NGS; 72.01% in TD; 71.05% in the combined cohorts) compared to between-person differences (28.90% in NGS; 27.99% in TD; 28.95% in the combined cohorts). Therefore, child age was disaggregated into a between-child variable ( $MChildAge_{i})$ and a within-child variable $\left(ChildAge_{ti}^{\left(PMC\right)}\right)$ . Specifically, child age was disaggregated by subtracting the age in months of the youngest child in their respective cohort from original values (NGS: 1.58 months; TD: 1.64 months, combined: 1.58 months), then the within-child variable was calculated by subtracting, for each child *i*, the mean of their own set of observations (i.e., person-mean centered).

Following the same procedure for caregiver age, analysis showed that most of the variance in mothers’ age was due to between-person differences (91.14% in NGS; 85.98% in TD; 90.27% in the combined cohorts), with much less within-person variance (8.86% in NGS; 14.02% in TD; 9.73% in combined cohorts). Given there was minimal within-person variance in caregiver age and the present study was primarily interested in between- and within-person effects of child age, caregiver age was not disaggregated. In addition, including within child and caregiver-age in the model as fixed predictors would have resulted in multicollinearity, further justifying why caregiver age was not disaggregated. Caregiver age was centered relative to the youngest participant from their corresponding group to facilitate the interpretation of the intercept (NGS: 22 years; TD: 23 years, combined: 22 years).

With regard to child sleep duration, ICC analyses revealed that greater variance was attributable to between-child differences compared to within-child differences in the NGS cohort (60.54% versus 39.46%, respectively) and combined cohorts (57.20% versus 42.80%, respectively). On the other hand, 19.25% and 80.75% of the variance in child sleep was attributed to between- and within-person differences in the TD cohort. Child sleep was disaggregated into a between-child variable $\left(MChildSleep_{i}\right)$ , which was calculated by subtracting the grand mean from all observations, and a within-child $\left(ChildSleep_{ti}^{\left(PMC\right)}\right)\;$ variable, calculated by subtracting for each child *i*, the mean of their own set of observations.

Following the unconditional models, a series of multilevel models were calculated for each outcome that included caregiver age, between- and within-person variables of child age, and between- and within-person variables of child sleep as fixed predictors, with a random effect of child age (i.e., Model A). A random effect of child sleep (i.e., Model B) was also considered (A detailed description of model selection and results in **Appendix E**), but three models estimating the random effect of child sleep did not converge for Model B. Only the Stress model in NGS estimating the random effect on child age did not converge for Model A. Thus, considering model fit and the number of models that converged, Model A was selected for the present analysis. The final model for each cohort (i.e., NGS, TD, and combined cohorts) was specified as noted below. The same model was repeated for anxiety and stress outcomes.


$$\begin{array}{c} {\text{Depression}}_{\text{ti}}=[\gamma_{00}+\gamma_{01}MCaregiverAge_{i}\\ +\gamma_{02}MChildAge_{i}+\gamma_{10}ChildAge_{ti}^{\left(PMC\right)}+\gamma_{03}MChildSleep_{i}+\gamma_{20}ChildSleep_{ti}^{\left(PMC\right)}\\ +\gamma_{04}(MChildSleep_{i}\times MChildAge_{i})+\gamma_{30}(ChildSleep_{ti}^{\left(PMC\right)}\\ \times ChildAge_{ti}^{\left(PMC\right)})]+[u_{0i}+u_{1i}ChildAge_{ti}^{\left(PMC\right)}]+r_{ti} \end{array}$$

Where Depression, Anxiety, and Stress for caregiver *i* at time *t*, is modeled as a function of the intercept level of self-reported mental health at the average level of all other predictors (γ_00_), the sample mean slope of the between-person effects of caregiver age ( $\gamma_{01}$ ), child age ( $\gamma_{02}$ ), and child sleep ( $\gamma_{03}$ ), the sample mean slope of the within-individual effects of child age ( $\gamma_{10}$ ) and child sleep ( $\gamma_{20}$ ), across the study, the interaction of child sleep and child age at the between- ( $\gamma_{04}$ ) and within-person level ( $\gamma_{30}$ ), person-specific error terms ( $u_{0i}$ ), which are person-specific deviations from the intercept and slope, the random effect of child age ( $u_{1i}$ ), and finally the time-specific residual error ( $r_{ti}$ ). In all, this model tested for the change in Depression, Anxiety, Stress with age (child and caregiver) and child sleep, as well as how the associations between child sleep and caregiver mental health may be moderated by child age over the course of the study. If moderations are significant, simple slopes will be conducted to examine the direction of effects.

## Results

Demographic characteristics of the sample are presented in **Table 2**. Data were included from a total of 193 caregivers, including 138 from the NGS cohort and 55 caregivers from the TD cohort. Child age varied from 1.58-108.41 months, and the sample was relatively balanced by child sex assigned at birth (46% female). In the total sample, 180 caregivers reported child racial and ethnic background: 170 were White, 4 were Asian, 1 was American Indian or Alaska Native, 1 was Black or African American, 1 was Native Hawaiian or Other Pacific Islander, 3 were Multiracial, and 4 identified as Hispanic.

**Table 2:** Demographic Characteristics and Raw Values of Variables of Interest by Cohort.

	NGS Cohort (N = 138)		TD Cohort (N = 55)		Combined Cohort (N =193)	
Female Children	69		20		89	
Male Children	69		35		104	
	Mean (*SD*)	Range	Mean (*SD*)	Range	Mean (*SD*)	Range
Caregiver Age (across assessments)	34.78 (4.77)	22 - 50	33.58 (3.84)	23 - 45	34.40 (4.53)	22 – 50
Child Age (across assessments)	40.91 (20.72)	1.58 - 108.41	36.71 (20.68)	1.64 - 92.51	39.60 (20.79)	1.58 - 108.41
Depression	2.71 (3.01)	0 - 20	2.73 (3.06)	0 - 17	2.71 (3.03)	0 – 20
Anxiety	2.03 (2.70)	0 - 15	1.58 (1.97)	0 -12	1.88 (2.50)	0 – 15
Stress	5.78 (3.77)	0 - 18	5.76 (3.43)	0 - 21	5.77 (3.67)	0 – 21
Child Sleep	644.39 (131.96)	270 - 1140	709.62 (88.86)	390 - 1080	666.40 (123.04)	270 – 1140
Child Sleep *Between-person*	636.57 (116.29)	330 - 862.50	711.44 (55.64)	570 - 877.50	659.16 (107.48)	330 - 877.50
Child Sleep *Within-person*	0.00 (70.79)	-210 - 277.50	0.00 (70.42)	-265 - 315	0.00 (70.62)	-265 – 315

NGS, Neurogenetic Syndrome; TD, Typically Developing; Combined, NGS and TD children. Child age values are in months, and caregiver age values are in years. Depression, Anxiety, and Stress scores are past-week symptoms from the DASS-21. Child Sleep values are collected in minutes, from the BISQ. Within-level values are person-mean centered.

Descriptive statistics for child sleep duration and for caregiver depression, anxiety, and stress scores are also presented in **Table 2**. As across groups, average scores were in the normal range on all three symptom scales, with scores varying from normal to severe. Average child sleep duration across observations was approximately 11 hours, with a wide range in child sleep duration (4.5 to 19 hours).

### Intraclass correlations of caregiver mental health outcomes

Findings from the unconditional models of caregiver mental health demonstrated that Depression score variance was related to both between-caregiver differences (57.63% in NGS; 59.16% in TD; 57.91% in combined cohorts) and within-caregiver variance (42.37% in NGS; 40.84% in TD; 42.09% in combined cohort). Similar findings for anxiety and stress score variances were observed at the between and within-caregiver levels, respectively, in the NGS cohort (i.e., anxiety: 57.55% versus 42.45%; stress: 64.02% versus 35.98%), TD cohort (i.e., anxiety: 50.47% versus 49.53%; stress: 60.39% versus 39.61%), and combined cohorts (i.e., anxiety: 56.11% versus 43.89%; stress: 63.18% versus 36.82%).

### Caregiver age, child age, and random effects

Next, the effect of caregiver age was modeled on depression, anxiety, and stress symptom severity. Results demonstrated a significant main effect of caregiver age on depression symptom severity among the NGS cohort (**Table 3**, $\gamma_{01\;}$ = .033, *se* = .010, *p* = .001) and the combined cohorts ( $\gamma_{01\;}$ = .025, *se* = .009, *p* = .005), indicating that older caregivers generally reported more severe depression symptoms scores across assessments compared to younger caregivers. On the contrary, no main effect of caregiver age was observed for depression scores in the TD cohort (**Table 3**), or across any cohorts for anxiety or stress scores (**Tables 4**, **5**).

**Table 3:** Between and Within-Person Effects of Child Sleep Duration and Depression Across Family Cohorts.

Depression	NGS Estimate (*SE*)	TD Estimate (*SE*)	Combined Estimate (*SE*)
*Fixed Effects*			
Intercept ( $\gamma_{00}$ )	2.824 (.769) *p<*.001***	4.237 (2.302) *p = .*072	2.990 (.699) *p<*.001***
Caregiver Age ( $\gamma_{01}$ )	.033 (.010) *p = .*001***	.003 (.017) *p = .*850	.025 (.009) *p = .*005**
Child Age ( $\gamma_{02}$ ) *Between-person level*	-.013 (.018) *p = .*477	-.113 (.043) *p = .*010**	-.021 (.016) *p = .*190
Child Age $\left(\gamma_{10}\right)$ *Within-person level*	-.003 (.002) *p = .*246	.0001 (.003) *p = .*983	-.002 (.002) *p* = .235
Child Sleep ( $\gamma_{03}$ ) *Between-person level*	-.002 (.001) *p* = .067	-.004 (.003) *p = .*182	-.002 (.001) *p = .*021*
Child Sleep ( $\gamma_{20}$ ) *Within-person level*	-.0003 (.0004) *p* = .489	.0002 (.0007) *p* = .759	-.0001 (.0004) *p = .*771
ChildAge*ChildSleep $\left(\gamma_{04}\right)$ *Between-person level*	.00001 (.00003) *p* = .607	.0002 (.00006) *p* = .004**	.00003 (.00002) *p = .*199
ChildAge*ChildSleep ( $\gamma_{30}$ ) *Within-person level*	-.00004 (.00002) *p* = .139	.00002 (.00004) *p* = .576	-.00001 (.00002) *p = .*628
*Error Variance*			
Intercept ( $u_{0i}$ )	.236 (.039) *p<*.001***	.314 (.071) *p<*.001***	.259 (.033) *p<*.001***
Child Age ( $u_{1i}$ ) *Within-person level*	.0002 (.0001) *p* = .015*	.0002 (.0001) *p* = .004**	.0001 (.0001) *p<.*001***
Residual ( $r_{ti}$ )	.173 (.016) *p<*.001***	.147 (.017) *p<*.001***	.165 (.012) *p<*.001***
*Model Fit*			
AIC	872.8	480.5	1276.1
BIC	884.5	488.5	1289.2

***p ≤.001, **p ≤.01, *p<.05, • marginally significant. Values based on SAS PROC Mixed. Entries show parameter estimates with standard errors in parentheses. Estimation Method =REML. Fixed Effects SE Method: Empirical. Containment degrees of freedom for all. Model Type = Unstructured. NGS model based on 471 observations nested within 138 caregivers. TD model is based on 247 observations nested within 55 caregivers. Mixed model based on 718 observations nested within 193 caregivers.

**Table 4:** Between and Within-Person Effects of Child Sleep Duration and Anxiety Across Family Cohorts.

Anxiety	NGS Estimate (*SE*)	TD Estimate (*SE*)	Combined Estimate (*SE*)
*Fixed Effects*			
Intercept ( $\gamma_{00}$ )	2.599 (.733) *p<*.001***	3.243 (1.672) *p = .*058	2.443 (.675) *p<*.001***
Caregiver Age ( $\gamma_{01}$ )	.006 (.010) *p = .*535	-.015 (.013) *p = .*254	.004 (.009) *p = .*610
Child Age ( $\gamma_{02}$ ) *Between-person level*	-.006 (.016) *p = .*718	-.039 (.041) *p = .*346	-.002 (.014) *p = .*901
Child Age $\left(\gamma_{10}\right)$ *Within-person level*	-.0003 (.002) *p = .*894	.001 (.003) *p = .*760	-.0001 (.002) *p = .*972
Child Sleep ( $\gamma_{03}$ ) *Between-person level*	-.002 (.001) *p* = .105	-.002 (.002) *p = .*351	-001 (.001) *p = .*158
Child Sleep ( $\gamma_{20}$ ) *Within-person level*	-.0004 (.0003) *p* = .241	-.0006 (.0005) *p* = .293	-.0004 (.0003) *p = .*165
ChildAge*ChildSleep $\left(\gamma_{04}\right)$ *Between-person level*	8.614E-6 (.00002) *p* = .711	.00005 (.00006) *p* = .403	-1.61E-7 (.00002) *p = .*994
ChildAge*ChildSleep ( $\gamma_{30}$ ) *Within-person level*	-.00001 (.00002) *p* = .624	.00003 (.00002) *p* = .275	4.838E-6 (.00002) *p = .*768
*Error Variance*			
Intercept ( $u_{0i}$ )	.194 (.032) *p<*.001***	.159 (.041) *p<*.001***	.182 (.025) *p<*.001***
Child Age ( $u_{1i}$ ) *Within-person level*	.0001 (.00004) *p* = .051•	.0002 (.00003) *p* = .219	.00004 (.00002) *p = .*051•
Residual ( $r_{ti}$ )	.150 (.013) *p*<.001***	.136 (.016) p<.001***	.146 (.010) p<.001***
*Model Fit*			
AIC	788.8	395.8	1101.0
BIC	800.5	403.9	1114.1

***p ≤.001, • marginally significant. Values based on SAS PROC Mixed. Entries show parameter estimates with standard errors in parentheses. Estimation Method =REML. Fixed Effects SE Method: Empirical. Containment degrees of freedom for all. Model Type = Unstructured. NGS model based on 471 observations nested within 138 caregivers. TD model is based on 247 observations nested within 55 caregivers. Mixed model based on 718 observations nested within 193 caregivers.

**Table 5:** Between and Within-Person Effects of Child Sleep Duration and Stress Across Family Cohorts.

Stress	NGS Estimate (*SE*)	TD Estimate (*SE*)	Combined Estimate (*SE*)
*Fixed Effects*			
Intercept ( $\gamma_{00}$ )	8.712 (3.481) *p = .*014*	26.300 (10.536) *p = .*016*	9.729 (3.193) *p = .*003**
Caregiver Age ( $\gamma_{01}$ )	.066 (.058) *p = .*258	-.067 (.073) *p = .*355	.050 (.050) *p = .*316
Child Age ( $\gamma_{02}$ ) *Between-person level*	.051 (.085) *p = .*555	-.516 (.232) *p = .*028*	.010 (.075) *p = .*894
Child Age $\left(\gamma_{10}\right)$ *Within-person level*	-.002 (.009) *p = .*864	.002 (.019) *p = .*898	-.003 (.008) *p = .*750
Child Sleep ( $\gamma_{03}$ ) *Between-person level*	-.006 (.005) *p* = .207	-.027 (.013) *p = .*041*	-.007 (.004) *p = .*107
Child Sleep ( $\gamma_{20}$ ) *Within-person level*	-.003 (.002) *p* = .086	-.003 (.003) *p* = .272	-.003 (.002) *p = .*036*
ChildAge*ChildSleep $\left(\gamma_{04}\right)$ *Between-person level*	-.00008 (.0001) *p* = .562	.0007 (.0003) *p* = .027*	-.00002 (.0001) *p = .*851
ChildAge*ChildSleep ( $\gamma_{30}$ ) *Within-person level*	-.00005 (.0001) *p* = .657	.00005 (.0001) *p* = .697	-.00002 (.0001) *p = .*841
*Error Variance*			
Intercept ( $u_{0i}$ )	7.707 (1.173) *p<.*001***	6.795 (1.576) *p<.*001***	8.110 (1.015) *p<.*001***
Child Age ( $u_{1i}$ ) *Within-person level*	Did not converge	.002 (.001) *p* = .053•	.001 (.001) *p = .*155
Residual ( $r_{ti}$ )	4.671 (.362) *p<.*001***	3.801 (.438) *p<*.001***	4.575 (.318) *p<*.001***
*Model Fit*			
AIC	2381.3	1225.5	3588.4
BIC	2390.1	1233.5	3601.5

***p ≤.001, **p<.01, *p<.05, • marginally significant. Values based on SAS PROC Mixed. Entries show parameter estimates with standard errors in parentheses. Estimation Method =REML. Fixed Effects SE Method: Empirical. Containment degrees of freedom for all. Model Type = Unstructured. NGS model based on 471 observations nested within 138 caregivers. TD model is based on 247 observations nested within 55 caregivers. Mixed model based on 718 observations nested within 193 caregivers.

With regard to child age, there was a main effect of child age at the between-family level only in the TD cohort for depression (**Table 3**, $\gamma_{02\;}$ = -.113, *se = .*043, *p* = .010) and stress scores (**Table 5**, $\gamma_{02}\;$ = -.516, *se = .*232, *p* = .028), indicating that caregivers with younger children generally reported more severe symptoms of depression and stress compared to caregivers with older children. No main effect of child age at the between-family level was observed for anxiety scores (**Table 4**). Similarly, across all cohorts, there were no main effects of child age at the within-family level across cohorts (**Tables 3**–**5**).

We next examined whether individual caregiver variability for each symptom category was greater than zero, both at the intercept level and across age. Here, the random intercepts were greater than zero for depression (**Table 3**), anxiety (**Table 4**), and stress (**Table 5**) in all cohorts. The random effect of age-related change in depression varied across individuals in each cohort (**Table 3**) but not for anxiety or stress scores (**Tables 4**, **5**). The random effect of age-related changes in stress scores for the NGS cohort did not converge.

### Child sleep duration and caregiver mental health outcome effects

We next examined our primary questions of whether child sleep predicated caregiver outcomes between dyads. As predicted, caregiver depression scores were associated with child sleep duration, but this was only observed at the between-caregiver level in the combined cohorts (**Table 3**, ${\text{ γ}}_{03}\;$ = -.002, *se* = .001, *p* = .021); results were similar but not statistically significant among the NGS and TD subgroups individually. Contrary to predictions, child sleep duration was not statistically associated with caregiver anxiety and stress scores across cohorts (**Table 4**) at the between caregiver-level. However, between caregiver-level effect of child sleep on stress was observed in the TD cohort (**Table 5**, ${\text{γ}}_{03}\;$ = -.027, *se* = .013, *p* = .041). Thus, dyads in which caregivers reported greater depression also tended to report worse child sleep, with the TD group also reporting this effect in relation to stress. No effects were observed in relation to anxiety.

Next, we examined the degree to which sleep predicted within-dyad changes in symptoms. Aligned with hypotheses, stress scores were associated with child sleep duration at the within-caregiver level in the combined cohorts (**Table 5**, $\gamma_{20}\;$ = -.003, *se = .*002, *p* = .036), suggesting that caregivers of children that slept less than usual at a specific occasion reported more severe stress symptoms. Although results were not statistically significant at the within-caregiver level among the NGS cohort and the TD cohort when examined separately, results show a similar direction of effects, suggesting a potential generalizable pattern across the full sample. Contrary to hypotheses, there were no main effects of child sleep on depression or anxiety scores across cohorts (**Tables 3**, **4**). Together, these results support our hypothesis that child sleep relates to short-term experiences of caregiver stress; however, this effect was not observed for caregiver depression or anxiety.

### Moderation effect of child age

Our exploratory analyses also examined how child age may moderate the association between child sleep and caregiver mental health, at both the between-caregiver level (i.e., comparing caregivers with older versus younger children) and at the within-caregiver level (i.e., comparing effects of child sleep as children aged over the course of the study). Analyses revealed that effects varied across age within the TD cohort when examining between-person effects of depression ( $\gamma_{04\;}$ = .0002, *se = .*00006, *p* =.004) and stress ( $\gamma_{04}\;$ = .0007, *se = .*0003, *p* = .027). These findings indicate that the strength of associations between child sleep duration and caregiver mental health varied across TD families based on child age, yet the associations were relatively consistent among the NGS families. To examine the direction of moderation of child age, simple slopes were computed for depression and stress across at the between-caregiver level in TD families (**Table 6**). A significant simple slope was observed at high levels of child age in depression only (i.e., 1+SD of Mean; *b* = .005, *se* = .002, *t* = 2.28, *p* = .024); slopes were not significant at low or average levels of child age (**Table 6**). No simple slopes were significant for stress (**Table 6**). These results suggest that among TD families, the association between greater depression and child sleep is primarily driven by experiences of families with older children.

**Table 6:** Simple slope analyses.

	*b*	*SE*	*t*	*p*
Depression				
1-SD	-.0001	.002	-.04	.971
Mean	.002	.002	1.20	.213
1+SD	.005	.002	2.28	.024*
Stress				
1-SD	-.012	.009	-1.25	.213
Mean	-.002	.009	-.210	.832
1+SD	.008	.011	.730	.469

*p<.05.

## Discussion

Child sleep problems affect the family system and can be particularly problematic for NGS families, in which both the child and caregiver are vulnerable to challenging outcomes. Due to these vulnerabilities, NGS dyads also offer a powerful model for understanding how predispositions to sleep challenges, mental health needs, and stress might change the ways in which family systems unfold over time. Past research has set the stage for this work by demonstrating that abnormal child sleep is generally associated with poorer caregiver outcomes in both NGS (26, 27) and non-NGS samples (12, 13). However, the ways in which these dynamics unfold over time are unclear, limiting understanding of how to best identify which dyads need support, and what types of support might be best for specific subgroups. For example, does sleep impact caregiver mental health at all ages, accumulate over time, or only matter when children are older? Are the associations between child sleep and caregiver mental health typically acute or long-lasting? Which caregiver outcomes are most impacted by child sleep? Answering these questions through longitudinal surveillance of NGS and non-NGS dyads can inform not only better understanding of how family dynamics relate to health, but also the provide insights into how – and when – supports might be offered to best improve outcomes within NGS communities and beyond.

To this end, the present study leveraged mixed effect modeling to characterize longitudinal associations between child sleep and caregiver well-being in a large, longitudinal cohort of young children with and without NGS. Due to the relatively high prevalence of sleep disruptions among NGS, this sample provided a unique opportunity to examine the consequences of disrupted child sleep on caregiver mental health, as well as how associations may generalize across the NGS and TD groups. The combined cohorts can be viewed conceptually as a ‘saturated’ sample to explore candidate associations between our variables of interest, which may not be apparent in non-clinical samples that have relatively low base rates of child sleep disruptions. Across cohorts, child age (collected in months) ranged from infancy to the school-age years, providing an opportunity to examine how associations differed at older ages, relative to younger ages when sleep problems are more common. Together, results indicated that child sleep and caregiver outcomes are indeed associated in this saturated sample, yet with complex patterns of timing and specificity. Here, we discuss key findings in the context of past literature and offer next steps for this line of work.

One of the primary findings of this study was detection of a *between-person* main effect of child sleep duration on caregiver depression symptom severity, such that caregivers of children that generally slept less reported greater depression symptom severity. A qualitatively similar (but non-significant) pattern was observed when NGS and TD sub-cohorts were observed separately, suggesting general levels of child sleep impairments may co-occur with caregiver depression similarly among NGS and TD families. However, the ways in which NGS and TD families experienced these associations across age differed: whereas child age did not moderate the strength of association between sleep and caregiver depression in the NGS group, this interaction was significant for the TD group, with families of older children exhibiting the greatest association. In other words, caregivers appeared most vulnerable to depression when sleep problems were being reported in older children. This specificity may indicate that for non-NGS families, a child’s sleep patterns may only produce depression when the child is at a developmental age in which sleep problems should generally be “normalized.” It could also be the case that when sleep problems are apparent in older children, caregivers have been managing cumulative impacts of poor sleep for greater duration of time, leading to experiences of depression.

This finding may be viewed within the broader context of a strong, well-established link between sleep problems and depression. During major depressive episodes, sleep is almost always disrupted, with insomnia being the most common clinical presentation (49). Critically, this association between sleep and depression is reciprocal: not only is sleep disruption a symptom of depression, but chronic sleep disruption also commonly precedes and increases risk for the subsequent onset of depression (50). In particular, chronic sleep disruption is thought to increase risk of depression in part through activation of the hypothalamic-pituitary-adrenal (HPA) axis, whereby persistent elevation in glucocorticoid levels may inhibit serotonergic signaling, impair the regulation of neuronal plasticity, and reduce hippocampal neurogenesis (for reviews, 51, 52). Consistent with this broader literature on major depression disorder, it is notable that the association between child sleep duration and caregiver depression symptom severity observed in the current study was only apparent at the between-person level (i.e., comparing families in which children exhibited shorter compared to longer sleep durations); in contrast, depression symptom severity did not systematically vary at the within-person level (i.e., within individual dyads according to whether the child had slept more or less). This between-person finding is consistent with the notion that chronic sleep disruption is an important risk factor for depression. Consequently, addressing chronic sleep has been suggested as a potential strategy for *preventing* depression (53). The current study highlights the importance of contextualizing sleep disruption within the family system when considering interventions to treat and prevent major depressive disorder.

Complementing the observed between-person association with caregiver depression symptoms, fluctuations in sleep *within* dyads were associated with concurrent fluctuations in stress symptom severity. That is, after taking into account each child’s typical sleep duration, a relative decrease in child sleep duration was associated with greater caregiver stress symptoms. Short- and long-term sleep disruptions are known to have distinct patterns of health consequences, with acute sleep disruption known to elicit transient stress responses, including activation of the sympathetic nervous system activation and elevated glucocorticoid levels (54, 55). Conversely, laboratory stress inductions also cause acute disruptions on next-night sleep, which are distinct from the effects of chronic stress on sleep (56). The self-reported stress scale used here captures past-week negative affect, with an emphasis on agitation, difficulty relaxing, and emotional sensitivity. Thus, the observed association with caregiver stress symptoms likely reflects a relatively short-term consequences of acute changes in child sleep duration. It may be of interest to extend this approach to disentangle the health implications of disturbed sleep at multiple time scales. Additionally, integrating direct measures of stress physiology could help clarify intermediate processes in this context.

Contrary to predictions, child sleep duration was not associated with caregiver anxiety symptom severity across any analyses or sub-cohorts. This is in contrast with the broader literature establishing that sleep disturbances are prominent in a wide range of anxiety and related disorders (57). One possible explanation for this null finding is that the anxiety scale used here emphasizes symptoms of physiological hyperarousal (i.e., panic-like symptoms), which are relatively unique to anxiety versus depression within the internalizing spectrum. Whereas panic disorder is associated with clinically significant sleep disturbance, this effect seems to be accounted for in part by anxiety sensitivity—anxious worry about physiological sensations—rather than overall panic symptom severity (58). Thus, it may be helpful to revisit the research question of child sleep and caregiver anxiety symptoms by incorporating scales that capture other features of anxiety, such as symptoms of worry, social anxiety, obsessive thinking, and generalized anxiety (59, 60).

The current study has several strengths as well as limitations. Strengths include the use of longitudinal framework and a relatively large sample size for NGS. To our knowledge, only one short-term longitudinal study on sleep, spanning 28 days, has been conducted in 16 children with AS (27). In the present study, data from 193 families yielded a total of 718 observations that span across several years, allowing us to examine this association over the course of time in a relatively large number of families impacted by NGS. Lastly, previous studies that examined caregiver mental health and child sleep in NGS communities assessed levels of stress. In the present study, we assessed dimensions of internalizing symptom severity, which revealed patterns of specificity that may help inform the nuances of how sleep impacts families, and potential targets and outcomes that should be addressed within sleep interventions.

There are also several limitations to consider. Firstly, our study used caregiver-report measures for both sleep and well-being variables. While the DASS-21 is a reliable, well-validated, and relatively short questionnaire designed for use in clinical and non-clinical samples, it is clinically limited in scope, capturing a subset of internalizing symptom phenotypes. Future studies might include more comprehensive measures of mental health symptom dimensions and stressors, which would help to clarify the relevant outcomes associated with disrupted sleep among caregivers. Secondly, given this a naturalistic study with rolling enrollment, a varying number of observations contributed to each family. Although staggered longitudinal studies are sound options for phenotyping low-incidence populations (61), more data would allow better understanding of how smaller fluctuations within dyads might predict outcomes. Additionally, the sample predominantly comprises families from similar racial and ethnic backgrounds, limiting its representation of the wider population. Future research should explore how the outcomes observed in this study apply across diverse racial and ethnic cohorts. Lastly, we focused our analyses on the key variable of sleep duration, to maximize consistency across the full age range within this cohort. To develop a more in-depth understanding of the relationship between child sleep, caregiver mental health, and child age, it may be helpful for future studies to explore if there are specific sleep phenotypes (e.g., night awakening frequency, sleep latency; 19) that are associated with worse caregiver mental health, especially given some sleep difficulties may be more or less common during a specific age range or for specific underlying conditions (23).

## Conclusions

Clinically significant disruptions in child sleep are common and have direct consequences on the health and well-being of caregivers. This is particularly true for NGS families due to the relative severity and persistence of child sleep disruptions, and in which both the child and caregiver are vulnerable to challenging outcomes. The current study builds upon the extant literature by leveraging data from an ongoing longitudinal study of NGS and non-NGS families, spanning infancy to school-age years. To capture the complexity of these data, a modeling approach was used to disentangle more temporally stable differences between dyads, fluctuations over time within-dyads, as well as potentially moderating effects of child age. A nuanced pattern of findings emerged, such that depressive symptoms were more severe among caregivers of children who slept less overall, whereas stress symptoms were more severe during periods of acute disruption in the child’s typical sleep duration. The link with depressive symptom severity was further shaped by child age among caregivers of typically developing children yet was relatively consistent with child age among NGS families. This pattern of results is broadly consistent with clinical and neuroendocrine literatures linking sleep disruptions to both short- and long-term health consequences. Child sleep and caregiver sleep are inextricably linked, and interventions that target sleep within a family systems context have the potential to improve health outcomes within NGS communities and beyond.

## Data availability statement

The raw data supporting the conclusions of this article will be made available by the authors, without undue reservation.

## Ethics statement

The studies involving humans were approved by Purdue Institutional Review Board. The studies were conducted in accordance with the local legislation and institutional requirements. Written informed consent for participation in this study was provided by the participants’ legal guardians/next of kin.

## Author contributions

KG-O: Writing – original draft, Conceptualization, Formal analysis. KM: Writing – review & editing, Formal analysis. DF: Writing – review & editing. BK: Funding acquisition, Project administration, Writing – review & editing, Conceptualization, Data curation, Formal analysis, Investigation, Methodology, Resources, Software, Supervision, Validation.
