# Supplementary material for: When they just don’t sleep: differential impacts of reduced child sleep on depression, anxiety, and stress among caregivers of children with and without neurogenetic syndromes
Source: Front Psychiatry. 2024 Apr 19;15:1352881. doi: 10.3389/fpsyt.2024.1352881 (PMC11067500; doi:10.3389/fpsyt.2024.1352881)
Supplement: Supplementary file 1 [file DataSheet_1.docx]

**Appendix A**

**Data Variables**

This appendix contains a key that outlines all the variables utilized for the analyses and for the SAS code. The variables below will be referenced from Appendix B to Appendix E.

Table 1

*Key Variables*

| Domain | Variable | Note |
| --- | --- | --- |
| Mental Health | dep_raw | Original depression values |
|  | anx_raw | Original anxiety values |
|  | stress_raw | Original stress values |
|  | depw | Winsorized depression values |
|  | anxw | Winsorized anxiety values |
|  | stressw | Winsorized stress values |
|  | sqrtDep | Variable depw transformed using square root |
|  | sqrtAnx | Variable anxw transformed using square root |
| Child Sleep | ChildSleep | Original child sleep duration values |
|  | ChildSleep_persMean | Between-child sleep duration |
|  | ChildSleep_persCtr | Within-child sleep  duration |
| Caregiver Variables | c_CaregiverAge | Centered caregiver age  (Original value minus youngest caregiver) |
|  | caregiver_id | Personal ID given to each caregiver |
| Child Variables | c_ChildAge | Centered child age  (Original value minus youngest child) |
|  | ChildAge_persMean | Between-child  age |
|  | ChildAge_persCtr | Within-child  age |

**Appendix** **B**

**Data Exploration and Transformation**

This appendix demonstrates the process involved in data exploration and the SAS code utilized to assess the variables of interest in the combined cohort. The same review process was used for the neurogenetic syndrome and typically developing cohort, independently. Within each cohort, we examined the distribution of variables. If variables were skewed (Table 2), then values were winsorized +/- 3 SD from the mean (Table 3). If skewness was still present, values were transformed using the square root method, which is a method commonly used and, unlike the log transformation, may be applied to zero values. If skewness had improved, values would not have undergone a square root transformation. In this case, the square root transformation resolved the problematic skewness for depression and anxiety values and improved data distribution (Table 4).

Table 2

*Combined cohorts* *Means and Standard Deviations of Original Values*

| Variable | Mean | SD | Min | Max | Skewness |
| --- | --- | --- | --- | --- | --- |
| dep_raw | 2.71 | 3.03 | 0 | 20.00 | 1.91 |
| anx_raw | 1.88 | 2.50 | 0 | 15.00 | 2.14 |
| stress_raw | 5.77 | 3.67 | 0 | 21.00 | 0.76 |
| ChildSleep | 666.40 | 123.04 | 270.00 | 1140.00 | -0.47 |

Table 3

*Combined cohorts* *Means and Standard Deviations of Winsorized Depression and Anxiety Variables*

| Variable | Mean | SD | Min | Max | Skewness |
| --- | --- | --- | --- | --- | --- |
| depw | 2.67 | 2.85 | 0 | 12.00 | 1.49 |
| anxw | 1.78 | 2.14 | 0 | 8.00 | 1.38 |
| stress_raw | 5.77 | 3.67 | 0 | 21.00 | 0.76 |
| ChildSleep | 666.40 | 123.04 | 270.00 | 1140.00 | -0.47 |

Table 4

*Combined cohorts Means and Standard Deviations of Square Root Depression and Anxiety Variable*

| Variable | Mean | SD | Min | Max | Skewness |
| --- | --- | --- | --- | --- | --- |
| sqrtDep | 1.79 | 0.67 | 1.00 | 3.61 | 0.83 |
| sqrtAnx | 1.56 | 0.58 | 1.00 | 3.00 | 0.86 |
| stress_raw | 5.77 | 3.67 | 0 | 21.00 | 0.76 |
| ChildSleep | 666.40 | 123.04 | 270.00 | 1140.00 | -0.47 |

*Example SAS Code (combined cohorts)*

*Plot Table for Variables of Interest;

**PROC** **MEANS** DATA=data N MEAN STDDEV MIN MAX VAR SKEW KURT;

VAR dep_raw anx_raw stress_raw ChildSleep;

**run**;

*Winsorized dep_raw and anx_raw will now be depw and anxw; **data** data_wins; set data;

if dep_raw = **.** then depw = **.** ;

else if dep_raw gt **12** then depw = **12**;

else if dep_raw lt -**12** then depw = -**12**;

else depw = dep_raw;

if anx_raw = **.** then anxw = **.** ; else if anx_raw gt **8** then anxw = **8**; else if anx_raw lt -**8** then anxw = -**8**; else anxw = anx_raw; **run**;

*Data check to review skewness;

**PROC** **MEANS** DATA=data_wins N MEAN STDDEV MIN MAX VAR SKEW KURT;

VAR depw anxw stress_raw ChildSleep;

**run**;

*Transformed depw and anxw using square root method; **DATA** data_wt; Set data_wins; sqrtDep=sqrt(depw+**1**);

sqrtAnx=sqrt(anxw+**1**);

**run**;

*Check skewness;

**PROC** **MEANS** DATA=data_wt n min mean max skew kurt;

VAR sqrtDep sqrtAnx stress_raw ChildSleep;

**run**;

**Appendix C**

**Centering Predictors**

In this appendix, I review how predictors were centered using SAS code. The ICCs for each predictor variable strongly suggested the need to disaggregate child sleep. Thus, for each predictor variable, I created a between- and within-person components (e.g., person mean, and person centered), yielding a total of 4 new variables (i.e., ChildSleep_persMean, ChildSleep_persCtr, ChildAge_perMean, ChildAge_persCtr).

*Example SAS Code*

**PROC** **SQL**;

CREATE TABLE PersMeanCent_data AS

SELECT *, mean(c_ChildAge) AS ChildAge_persMean, mean(ChildSleep) AS ChildSleep_persMean, c_ChildAge - mean (c_ChildAge) AS ChildAge_persCtr,

ChildSleep - mean(ChildSleep) AS ChildSleep_persCtr

FROM data_wt

GROUP BY caregiver_id;

**QUIT**;

**Appendix D**

**Intraclass Correlation Coefficient Calculations**

In this appendix, I provide an example SAS code for the unconditional models, which were used to calculate the ICCs for each cohort, independently. ICCs were calculated for each variable of interest, including age, child sleep, and mental health outcomes (e.g., depression, anxiety, and stress) to determine the change in between and within-caregiver variance when adding each variable in the model.

*Example SAS Code*

*ICCs for sqrtDep ;

**proc** **mixed** covtest noclprint data= PersMeanCent_data method=reml;

class caregiver_id;

model sqrtDep = /solution ddfm = KENWARDROGER2;;

random intercept / sub=caregiver_id type=un;;

**run**;

**Appendix E**

**Model Determination**

In this appendix, I review how the model for the current study was selected. SAS code is also included below. During the process of model specification, I began with 2 models to assess model convergence and model fit per the AIC and BIC. In Model A, the following fixed predictor variables were included: between-caregiver age, between-child age, within-child age, between-child sleep, and within-child sleep. Model A included a random effect of within-child age. Model B consisted of the same fixed predictors, but a random effect of within-child sleep duration was included in Model B instead of the random effect of within-child age. Upon the examination of the models, I decided to utilize Model A. Model B did not converge when assessing symptoms of anxiety, despite the use of the “EMPIRICAL” option (an estimation procedure that uses robust standard errors and decreases bias associated with the violation of assumptions of normality of the residuals). Also, Model A had relatively better model fit, per the AIC and BIC, and is also more aligned with our primary research questions, which focus heavily on child age. Below you will find mixed effects models estimated for each mental health outcome across all cohorts and an example of SAS code used for one of the models.

*Example SAS Code*

**proc** **mixed** covtest noclprint data=PersMeanCent_data method=reml EMPIRICAL;

class caregiver_id;

model sqrtDep = c_CaregiverAge ChildAge_persMean ChildAge_persCtr ChildSleep_persMean ChildSleep_persCtr ChildAge_persMean*ChildSleep_persMean ChildAge_persCtr*ChildSleep_persCtr /solution ddfm = CONTAIN;;

random intercept ChildAge_persCtr / sub=caregiver_id type=un;

**run**;

| Table 5  *Between and Within-Person Effects of Child Sleep Duration on Depression Across Groups: Model A and B* | | | | | | |
| --- | --- | --- | --- | --- | --- | --- |
|  | Neurogenetic Syndrome Cohort | | Typically Developing  Cohort | | Combined Cohorts | |
|  | Model A:  Random Effect Child Age | Model B:  Random Effect Child Sleep | Model A:  Random Effect Child Age | Model B:  Random Effect Child Sleep | Model A:  Random Effect Child Age | Model B:  Random Effect Child Sleep |
| ***Fixed Effects*** |  |  |  |  |  |  |
| Intercept ($\gamma_{00}$) | 2.824  (.769)  *p* <.001*** | 2.806  (.769)  *p* <.001*** | 4.237  (2.302)  *p =* .072 | 4.063  (2.585)  *p =* .122 | 2.990  (.699)  *p* <.001*** | 2.983  (.729)  *p <*.001*** |
| Caregiver Age ($\gamma_{01}$) | .033  (.010)  *p =* .001*** | .031  (.010)  *p =* .002** | .003  (.017)  *p =* .850 | .003  (.023)  *p =* .889 | .025  (.009)  *p = .*005** | .025  (.009)  *p =* .006** |
| Child Age ($\gamma_{02})$  *Between-person level* | -.013  (.018)  *p =* .477 | -.012  (.019)  *p* = .534 | -.113  (.043)  *p =* .010** | -.106  (.064)  *p =* .108 | -.021  (.016)  *p =* .190 | -.021  (.018)  *p =* .243 |
| Child Age ${(\gamma}_{10})$  *Within-person level* | -.003  (.002)  *p =* .246 | -.003  (.002)  *p =* .112 | .0001  (.003)  *p =* .983 | -.0004  (.003)  *p =* .912 | -.002  (.002)  *p* = .235 | -.003  (.002)  *p =* .115 |
| Child Sleep ($\gamma_{03}$)  *Between-person level* | -.002  (.001)  *p* = .067 | -.002  (.001)  *p =* .082 | -.004  (.003)  *p =* .182 | -.004  (.003)  *p =* .298 | -.002  (.001)  *p = .*021* | -.002  (.001)  *p =* .036* |
| Child Sleep ($\gamma_{20}$)  *Within-person level* | -.0003  (.0004)  *p* = .489 | -.0006  (.0005)  *p =* .196 | .0002  (.0007)  *p* = .759 | -.0001  (.0008)  *p =* .926 | -.0001  (.0004)  *p =* .771 | -.0003  (.0004)  *p* = .372 |
| ChildAge*ChildSleep $(\gamma_{04})$  *Between-person level* | .00001  (.00003)  *p* = .607 | .00001  (.00003)  *p =* .680 | .0002  (.00006)  *p* = .004** | .0002  (.00001)  *p =* .081 | .00003  (.00002)  *p = .*199 | .00003  (.00003)  *p* = .273 |
| ChildAge*ChildSleep ($\gamma_{30}$)  *Within-person level* | -.00004  (.00002)  *p* = .139 | -.00006  (.00003)  *p =* .031* | .00002  (.00004)  *p* = .576 | 2.582E-6  (.00003)  *p =* .933 | -.00001  (.00002)  *p = .*628 | -.00003  (.00002)  *p* = .135 |
| ***Error Variance*** |  |  |  |  |  |  |
| Intercept ($u_{0i}$) | .236  (.039)  *p* <.001*** | .229  (.036)  *p* <.001*** | .314  (.071)  *p* <.001*** | .307  (.071)  *p* <.001*** | .259  (.033)  *p* <.001*** | .254  (.033)  *p <*.001*** |
| Child Age ($u_{1i}$)  *Within-person level* | .0002  (.0001)  *p* = .015* | 5.211E-6  (1.875E-6)  *p =* .003** | .0002  (.0001)  *p* = .004** | 4.129E-6  (2.273E-6)  *p =* .035* | .0001  (.0001)  *p <* .001*** | .4.411E-6  (1.372E-6)  *p <* .001*** |
| Residual ($r_{ti}$) | .173  (.016)  *p* <.001*** | 0.166  (.015)  *p* <.001*** | .147  (.017)  *p* <.001*** | .172  (.019)  *p* <.001*** | .166  (.012)  *p* <.001*** | .170  (.012)  *p* <.001*** |
| ***Model Fit*** |  |  |  |  |  |  |
| AIC | 872.8 | 864.7 | 480.5 | 489.5 | 1276.1 | 1274.0 |
| BIC | 884.5 | 876.5 | 488.5 | 497.6 | 1289.2 | 1287.1 |
| Note: *** p ≤ .001, ** p ≤ .01, * p < .05, ● marginally significant. Values based on SAS PROC Mixed. Entries show parameter estimates with standard errors in parentheses. Estimation Method =REML. Fixed Effects SE Method: Empirical. Containment degrees of freedom for all. Model Type = Unstructured. NGS model based on 471 observations nested within 138 caregivers. TD model is based on 247 observations nested within 55 caregivers. Mixed model based on 718 observations nested within 193 caregivers. | | | | | | |

| Table 6  *Between and Within-Person Effects of Child Sleep Duration on Anxiety Across Groups: Model A and B* | | | | | | |
| --- | --- | --- | --- | --- | --- | --- |
|  | Neurogenetic Syndrome Cohort | | Typically Developing  Cohort | | Combined Cohorts | |
|  | Model A:  Random Effect Child Age | Model B:  Random Effect Child Sleep | Model A:  Random Effect Child Age | Model B:  Random Effect Child Sleep | Model A:  Random Effect Child Age | Model B:  Random Effect Child Sleep |
| ***Fixed Effects*** |  |  |  |  |  |  |
| Intercept ($\gamma_{00}$) | 2.599  (.733)  *p* <.001*** | 2.616  (.712)  *p* <.001*** | 3.243  (1.672)  *p =* .058 | 3.768  (1.876)  *p =* .050● | 2.443  (.675)  *p* <.001*** | 2.455  (.622)  *p <*.001*** |
| Caregiver Age ($\gamma_{01}$) | .006  (.010)  *p =* .535 | .006  (.010)  *p =* .491 | -.015  (.013)  *p =* .254 | -.016  (.016)  *p =* .332 | .004  (.009)  *p =* .610 | .004  (.008)  *p* = .577 |
| Child Age ($\gamma_{02})$  *Between-person level* | -.006  (.016)  *p =* .718 | -.006  (.018)  *p =* .729 | -.039  (.041)  *p =* .346 | -.052  (.047)  *p =* .273 | -.002  (.014)  *p =* .901 | -.002  (.015)  *p* = .890 |
| Child Age ${(\gamma}_{10})$  *Within-person level* | -.0003  (.002)  *p =* .894 | -.0004  (.002)  *p =* .852 | .001  (.003)  *p =* .760 | .0005  (.003)  *p =* .860 | -.0001  (.002)  *p =* .972 | -.0001  (.002)  *p* = .935 |
| Child Sleep ($\gamma_{03}$)  *Between-person level* | -.002  (.001)  *p* = .105 | -.002  (.001)  *p =* .109 | -.002  (.002)  *p =* .351 | -.003  (.002)  *p =* .275 | -001  (.001)  *p =* .158 | -.001  (.001)  *p* =.143 |
| Child Sleep ($\gamma_{20}$)  *Within-person level* | -.0004  (.0003)  *p* = .241 | -.0005  (.0004)  *p =* .210 | -.0006  (.0005)  *p* = .293 | -.0008  (.0006)  *p =* .125 | -.0004  (.0003)  *p =* .165 | -.0004  (.0003)  *p* = .222 |
| ChildAge*ChildSleep $(\gamma_{04})$  *Between-person level* | 8.614E-6  (.00002)  *p* = .711 | 8.998E-6  (.00003)  *p =* .742 | .00005  (.00006)  *p* = .403 | .00007  (.00007)  *p =* .300 | -1.61E-7  (.00002)  *p =* .994 | 9.352E-9  (.00002)  *p* = 1.00 |
| ChildAge*ChildSleep ($\gamma_{30}$)  *Within-person level* | -.00001  (.00002)  *p* = .624 | -.00002  (-.00002)  *p =* .434 | .00003  (.00002)  *p* = .275 | .00003  (.00003)  *p =* .324 | 4.838E-6  (.00002)  *p =* .768 | 2.053E-6  (.00002)  *p* =. 908 |
| ***Error Variance*** |  |  |  |  |  |  |
| Intercept ($u_{0i}$) | .194  (.032)  *p <*.001*** | .193  (.031)  *p <*.001*** | .159  (.041)  *p <*.001*** | .153  (.039)  *p* <.001*** | .182  (.025)  *p <*.001*** | .176  (.024)  *p <*.001*** |
| Child Age ($u_{1i}$)  *Within-person level* | .0001  (.00004)  *p* = .051● | 2.594E-6  (1.694E-6)  *p =* .063 | .0002  (.00003)  *p* = .219 | Did not  converge | .00004  (.00002)  *p =* .051● | Did not converge |
| Residual ($r_{ti}$) | .150  (.013)  *p* <.001*** | .148  (.014)  *p* <.001*** | .136  (.016)  *p* <.001*** | .142  (.015)  *p* <.001*** | .146  (.010)  *p* <.001*** | .150  (.011)  *p <.*001*** |
| ***Model Fit*** |  |  |  |  |  |  |
| AIC | 788.8 | 789.1 | 395.8 | 395.7 | 1101.0 | 1104.0 |
| BIC | 800.5 | 800.8 | 403.9 | 401.7 | 1114.1 | 1117.0 |
| Note: *** p ≤ .001, ** p ≤ .01, * p < .05, ● marginally significant. Values based on SAS PROC Mixed. Entries show parameter estimates with standard errors in parentheses. Estimation Method =REML. Fixed Effects SE Method: Empirical. Containment degrees of freedom for all. Model Type = Unstructured. NGS model based on 471 observations nested within 138 caregivers. TD model is based on 247 observations nested within 55 caregivers. Mixed model based on 718 observations nested within 193 caregivers. | | | | | | |

| Table 7  *Between and Within-Person Effects of Child Sleep Duration on Stress Across Groups: Model A and B* | | | | | | |
| --- | --- | --- | --- | --- | --- | --- |
|  | Neurogenetic Syndrome Cohort | | Typically Developing  Cohort | | Combined Cohorts | |
|  | Model A:  Random Effect Child Age | Model B:  Random Effect Child Sleep | Model A:  Random Effect Child Age | Model B:  Random Effect Child Sleep | Model A:  Random Effect Child Age | Model B:  Random Effect Child Sleep |
| ***Fixed Effects*** |  |  |  |  |  |  |
| Intercept ($\gamma_{00}$) | 8.712  (3.481)  *p =* .014* | 8.405  (4.360)  *p =* .056● | 26.300  (10.536)  *p =* .016* | 26.552  (11.898)  *p =* .030* | 9.729  (3.193)  *p =.*003** | 9.128  (3.961)  *p* = .022* |
| Caregiver Age ($\gamma_{01}$) | .066  (.058)  *p =* .258 | .069  (.056)  *p =* .226 | -.067  (.073)  *p =* .355 | -.024  (.104)  *p =* .818 | .050  (.050)  *p =* .316 | .056  (.050)  *p* = .266 |
| Child Age ($\gamma_{02})$  *Between-person level* | .051  (.085)  *p =* .555 | .061  (.108)  *p =* .569 | -.516  (.232)  *p =* .028* | -.533  (.299)  *p =* .080 | .010  (.075)  *p =* .894 | .024  (.096)  *p* = .799 |
| Child Age ${(\gamma}_{10})$  *Within-person level* | -.002  (.009)  *p =* .864 | -.005  (.010)  *p =* .609 | .002  (.019)  *p =* .898 | -.006  (.016)  *p =* .679 | -.003  (.008)  *p =* .750 | -.006  (.009)  *p* = .521 |
| Child Sleep ($\gamma_{03}$)  *Between-person level* | -.006  (.005)  *p* = .207 | -.006  (.006)  *p =* .374 | -.027  (.013)  *p =* .041* | -.028  (.016)  *p =* .077 | -.007  (.004)  *p =*.107 | -.006  (.006)  *p* = .285 |
| Child Sleep ($\gamma_{20}$)  *Within-person level* | -.003  (.002)  *p* = .086 | -.005  (.002)  *p =* .031* | -.003  (.003)  *p* = .272 | -.005  (.003)  *p =* .112 | -.003  (.002)  *p = .*036* | -.004  (.002)  *p* = .023* |
| ChildAge*ChildSleep $(\gamma_{04})$  *Between-person level* | -.00008  (.0001)  *p* = .562 | -.0001  (.0002)  *p =* .569 | .0007  (.0003)  *p* = .027* | .0007  (.0005)  *p =* .082 | -.00002  (.0001)  *p =* .851 | -.00004  (.0001)  *p* = .762 |
| ChildAge*ChildSleep ($\gamma_{30}$)  *Within-person level* | -.00005  (.0001)  *p* = .657 | -.00008  (.0001)  *p =* .549 | .00005  (.0001)  *p* = .697 | .00007  (.0002)  *p =* .616 | -.00002  (.0001)  *p = .*841 | -.00003  (.0001)  *p* = .756 |
| ***Error Variance*** |  |  |  |  |  |  |
| Intercept ($u_{0i}$) | 7.707  (1.173)  *p <.*001*** | 7.976  (1.167)  *p <.*001*** | 6.795  (1.576)  *p <.*001*** | 6.223  (1.562)  *p <.*001*** | 8.110  (1.015)  *p <.*001*** | 7.950  (.982)  *p <.*001*** |
| Child Age ($u_{1i}$)  *Within-person level* | Did not  converge | .0001  (.0001)  *p =* .020* | .002  (.001)  *p* = .053● | Did not  converge | .001  (.001)  *p =* .155 | .00004  (.00003)  *p* = .073 |
| Residual ($r_{ti}$) | 4.671  (.362)  *p <.*001*** | 4.064  (.367)  *p <.*001*** | 3.801  (.438)  *p* <.001*** | 4.275  (.437)  *p <.*001*** | 4.575  (.318)  *p* <.001*** | 4.484  (.311)  *p <*.001*** |
| ***Model Fit*** |  |  |  |  |  |  |
| AIC | 2381.3 | 2374.2 | 1225.5 | 1226.6 | 3588.4 | 3584.0 |
| BIC | 2390.1 | 2385.9 | 1233.5 | 1232.7 | 3601.5 | 3597.1 |
| Note: *** p ≤ .001, ** p ≤ .01, * p < .05, ● marginally significant. Values based on SAS PROC Mixed. Entries show parameter estimates with standard errors in parentheses. Estimation Method =REML. Fixed Effects SE Method: Empirical. Containment degrees of freedom for all. Model Type = Unstructured. NGS model based on 471 observations nested within 138 caregivers. TD model is based on 247 observations nested within 55 caregivers. Mixed model based on 718 observations nested within 193 caregivers. | | | | | | |
